# The Effect of Persuasive Messages in Promoting Home-Based Physical Activity During COVID-19 Pandemic

**DOI:** 10.3389/fpsyg.2021.644050

**Published:** 2021-04-01

**Authors:** Valentina Carfora, Patrizia Catellani

**Affiliations:** Catholic University of the Sacred Heart, Milan, Italy

**Keywords:** message frame, home-based physical activity, COVID-19, lockdown, exercising at home, psychosocial

## Abstract

We tested the plausibility of a persuasion model to understand the effects of messages framed in terms of gain, non-loss, loss, and non-gain, and related to the physical, mental and social consequences of doing physical activity at home during the lockdown restrictions. 272 Italian participants responded to a questionnaire on their attitude and intention at Time 1, frequency of past behavior, and self-efficacy related to exercising at home. Then, participants were randomly assigned to four different message conditions: (a) gain messages focused on the positive outcomes associated with doing physical activity at home; (b) non-loss messages focused on the avoided negative outcomes associated with doing physical activity at home; (d) loss messages focused on the negative outcomes associated with not doing physical activity at home; (c) non-gain messages focused on the missed positive outcomes associated with not doing physical activity at home. After reading the messages, participants answered a series of questions regarding their perception of threat and fear, their evaluation of the messages, and their attitude and intention toward exercising at home at Time 2. Using multigroup structural equation modeling, we compared message conditions, and tested whether the effects of the messages on attitude and intention at Time 2 were mediated by message-induced threat, message-induced fear, and message evaluation. Results showed that the perception of the messages as not threatening was the key point to activate a positive evaluation of the recommendation. The highest persuasive effect was observed in the case of the non-loss frame, which did not threaten the receivers, triggered a moderated fear and, in turn, activated a positive evaluation of the recommendation, as well as higher attitude and intention to do home-based physical activity at Time 2. Overall, these results advance our comprehension of the effects of message framing on receivers' attitudes and intentions toward home-based physical activity.

## Introduction

The Coronavirus (Covid-19) appeared in December 2019 in China (Wuhan) and the infection rapidly spread throughout the world. Three months later, Covid-19 became a worldwide pandemic with more than 1,728,878 cases confirmed on December 07th, 2020 and 60,078 deaths in Italy (Coronavirus Statistiques, 2020). At the beginning of the pandemic, Italy was one of the most seriously affected countries and, on March 08th, 2020, the Italian Government implemented extraordinary measures to limit viral transmission, including social and physical distancing measures, lockdown of industry, school, and overall social life. Although these measures have proven to be the best option to reduce the rapid spread of infections, this has produced collateral effects on other dimensions, determining a radical change in the lifestyle of the Italian population (Cancello et al., 2020; Cavallo et al., 2020; Odone et al., 2020).

Requiring a large-scale behavior change, the COVID-19 pandemic has raised the importance to apply the insights from psycho-social and behavioral sciences to promote people's adherence to the recommendations of epidemiologists and public health experts. In particular, this event has highlighted the relevance of the use of persuasive communication to educate people around preventive health behaviors. Evidence for the effectiveness of persuasive messages to promote health behaviors has been built over the last decades (e.g., Gallagher and Updegraff, 2012), but it has also received confirmation during the COVID-19 pandemic. Many scholars have shown that persuasive messages can facilitate policy-makers to promote prevention behaviors during a global public health crisis, and have confirmed the importance of finding efficient messages, as an easy and potentially scalable public intervention (e.g., Bilancini et al., 2020; Capraro and Barcelo, 2020; Heffner et al., 2020; Jordan et al., 2020; Lunn et al., 2020; Søraa et al., 2020). However, there do not seem to be any studies specifying how to formulate persuasive messages to promote home-based physical activity during the lockdown, even if one of the major changes regarded a reduction in the level of physical activity and sport, due to the closure of gyms, stadiums, pools, dance and fitness studios, physiotherapy centers, parks, and playgrounds (Serafini et al., 2020).

During the COVID-19 pandemic, many health communication practitioners designed persuasive messages to reduce the negative effects of the imposed restrictions on physical and mental health, such as the unhealthy consequences of sedentary behavior. For this reason, the evaluation of how persuasive messages impact on people' behavior appears as more necessary than ever. Even if health communication campaigns are often effective at changing individuals' behaviors (Anker et al., 2016), in some cases they can also have a “boomerang effect” that results in receivers adopting behaviors opposite to the health recommendation (Byrne and Hart, 2009). This counterproductive effect may be generated when receivers perceive health messages as too fearful or threatening.

To overcome this possible counterproductive effect of health communication, in the present study we aimed at clarifying the role of threat and fear induced by messages promoting home-based physical activity during the COVID-19 outbreak. We specifically tested whether differently framed messages can differently involve receivers both cognitively and emotionally, thus influencing their attitude and intention toward home-based physical activity. Generally, health guidelines recommend that all adults should engage in at least 150–300 min a week of moderate-intensity exercise (Piercy et al., 2018) and this recommendation was even more valid during the quarantine for at least two reasons. First, regular exercise may reduce the risk of acute respiratory distress syndrome, a major cause of death in patients COVID-19 (University of Virginia Health System, 2020). Second, regular exercise is associated with emotional resilience to stress (Childs and de Wit, 2014), one of the positive psychological responses observed during times of pandemics (Taylor et al., 2020). However, physical activity guidelines alone are unlikely to increase physical activity levels of the population (Milton et al., 2020). Appropriate and effective communication is key to maximizing the impact of such guidelines. In the present study, we tested whether differently framed messages can differently involve receivers both cognitively and emotionally, influencing their attitude and intention toward indoor home-based physical activity.

### Message Framing

Under given conditions, persuasive messages stimulate attitude change, and consequent change in intention and behavior regarding physical activity (e.g., Ajzen, 1991; Eagly and Chaiken, 1993; Petty and Cacioppo, 2012; Petty and Briñol, 2015). Research has shown that the persuasive effect depends, at least in part, on how message recommendations are framed (Davis, 1995; Chong and Druckman, 2007; Spence and Pidgeon, 2010). For example, recommendation messages can differ as to their valence frame, that is, their stress on either the positive or the negative consequences of a given behavior (e.g., Rothman et al., 2006). While a positively framed message presents the positive outcomes associated with the implementation of the recommended behavior, a negatively framed message presents the negative outcomes associated with not performing the recommended behavior.

Existing evidence suggests that positively framed messages regarding various outcomes of physical activity are more effective than negatively framed messages (e.g., Jones et al., 2003; Kozak et al., 2013; for a review: Williamson et al., 2020). For example, found that gain-framed messages were more effective in increasing participants' action planning regarding physical activity. Similarly, van't Riet et al. (2010) showed that gain-framed messages were more persuasive than loss-framed messages in advocating physical activity.

Messages can be framed not only as regards their gain or loss valence, but also as regards a further level of framing, namely, the *outcome sensitivities level* of message framing (Cesario et al., 2013). According to this framing level, gain-framed messages can be further diversified in messages focused on actual *gain*, when they describe the presence of positive outcomes (e.g., ≪If you eat well, you will improve your health≫), and messages focused on *non-loss*, when they focus on the absence of negative outcomes (e.g., ≪If you eat well, you will avoid damaging your health≫). Likewise, loss-framed messages can be further diversified in messages focused on actual *loss*, when they emphasize the presence of negative outcomes (e.g., ≪If you eat badly, you will damage your health≫) and messages focused on *non-gain*, when they focus on the absence of positive outcomes (e.g., ≪If you eat badly, you will miss the opportunity to improve your health≫).

The different effects of gain, non-loss, non-gain and loss messages have been studied in communication advocating different types of healthy behavior (e.g., Dijkstra et al., 2011; Carfora et al., 2020). For example, Carfora et al. (2021) considered the aforementioned four types of messages to promote healthy eating and showed that they induce different message evaluations, which in turn influences attitude and intention, via a cognitive or emotional elaboration. Besides, Carfora et al. (2020) showed that gain and non-loss messages activate an integrated emotional and cognitive processing of the health recommendation, while loss and non-gain messages mainly activate emotional shortcuts toward attitude and intention. Finally, the differential influence of these four message frames on attitude and intention has been shown to vary according to some baseline psychosocial features, such as self-efficacy (e.g., Di Massimo et al., 2019; Carfora et al., 2020).

To the best of our knowledge, so far research on the promotion of physical activity has ignored the distinction among gain, non-loss, non-gain, and loss message framing. For example, Strachan et al. (2020) compared the effects of gain- and loss-framed messages to promote physical activity, including non-loss outcomes in the gain-framed messages (e.g., reduced risk of diseases, less anxiety) and non-gain outcomes in the loss-framed messages (e.g., decreased attractiveness through reduced muscle tone). To move further in the comprehension of the factors that may underly the different effectiveness of the four types of messages, in the present study we submitted these messages to different groups of participants and explored the reactions receivers have when they are exposed to these messages. We aimed to assess the cognitive and emotional mechanisms underlying message influence on attitude and intention toward increased home-based physical activity, as well as possible differences in the role played by these mechanisms according to the message type. Below, the cognitive and emotional mechanisms investigated in the study are discussed in detail.

### Message-Induced Threat

The basic premise of persuasion models is that attitude and intention changes depend upon the likelihood that a persuasive issue or argument will be positively evaluated by the receiver (Petty and Cacioppo, 1986; Eagly and Chaiken, 1993). Message evaluation has a direct effect on receivers' attitude and intention toward the behavior recommended in the message (e.g., Cauberghe et al., 2009; Fernando et al., 2016), and this effect has been demonstrated also when the recommended behavior regards physical activity (Jones et al., 2003). In the present study, we moved from the assumption that the effect of message framing on attitude and intention would at least partially depend on how differently framed messages would be evaluated.

One of the aspects influencing the evaluation of a health recommendation message is the extent to which receivers perceive the message as threatening. According to psychological reactance theory, when individuals feel that someone or something is pressuring them to accept a certain view or attitude that limit their freedom, they activate psychological reactance to restore the lost freedom (Brehm and Brehm, 1981). Since recommendation messages in health communication aim to shape, reinforce, or change attitudes and behaviors, this attempt can be therefore perceived as a threat to freedom (Shen, 2015). As regards physical activity, receivers may perceive a message recommending it as threatening. Thus, they may not process it accurately and instead respond defensively (Liberman and Chaiken, 1992), for example downplaying its recommendation (Falk et al., 2015; Howe and Krosnick, 2017). According to self-affirmation theory (Steele, 1988; Sherman and Cohen, 2006), people may react defensively to threatening messages because they seek to maintain self-integrity, i.e., a perception of being capable of controlling important outcomes. When self-integrity is threatened, people seek to protect or restore it, often rejecting or denigrating threatening information (Cohen and Sherman, 2014). Thus, exposure to physical activity messages may threaten the self-integrity of individuals (McQueen and Klein, 2006; Jessop et al., 2014). In this threatened state, the ability to process a message recommending increased physical activity may be compromised because people, in order to maintain self-integrity, may question or reject the validity of the recommendation, or direct attention away from it (Sherman, 2013; Strachan et al., 2020). However, so far, no research has analyzed how perceived threat after exposure to differently framed messages recommending physical activity may negatively influence receivers' attitudes and intentions.

### Message-Induced Fear

Receivers' processing and evaluation of health recommendation messages is also influenced by affective responses triggered by messages themselves (e.g., Gross and D'ambrosio, 2004; Dillard and Nabi, 2006; Peters et al., 2006; Kühne et al., 2015). This is also the case when the recommendation message regards physical activity (Michalovic et al., 2018), and fear is one of the emotions that is more likely to influence the evaluation and the effect of a health recommendation message. There is overwhelming evidence of a positive fear–persuasion relationship (e.g., King and Reid, 1990; LaTour and Rotfeld, 1997; Dillard and Anderson, 2004). Messages evoking fear lead people to rely on systematic processing, which in turn stimulates many issue-relevant thoughts, and thus a positive message evaluation (e.g., Meijnders et al., 2001; Slater et al., 2002; Meyers-Levy and Maheswaran, 2004). Consistently, a long history of research has led to the general conclusion that messages inducing fear are more effective than those that do not (for a meta-analysis, see De Hoog et al., 2007), and the investigated effects include attitude and intention change toward a variety of health-related behaviors (for a meta-analysis, see Tannenbaum et al., 2015). Once said that, some research has also shown that messages inducing fear can be counterproductive. Fear can induce people to enact defensive strategies to reduce the potential emotional distress associated with the message. These strategies can include directing attention away from the message, reinterpreting or disregarding it (Witte, 1992; Ruiter et al., 2001). In the case of differently framed messages recommending increased physical activity, the different frames are likely to trigger different levels of fear in the receivers. However, we lack empirical evidence of whether and how far this is the case, as well as of related effects on attitude and intention.

Starting from the above, in the present study we examined whether and how far physical activity recommendations framed as gain, non-loss, non-gain, or loss (i.e., varying according to the outcome sensitivities level of message framing) would be perceived as threatening or induce fear. We also examined whether perceived threat or fear would have an impact on message evaluation toward home-based physical activity. Self-efficacy, frequency of past behavior and habit to exercise regularly have found to be some of the main predictors of physical activity, in general and also during the lockdown due to the COVID-19 pandemic (Carriedo et al., 2020; Rhodes et al., 2020), in addition to attitude and intention (Kaushal and Srivastava, 2020). Consistently, in the present research we tested the effects of differently framed messages not only on message evaluation, but also on attitude and intention toward home-based physical activity. Finally, we controlled for the independent effects of self-efficacy and frequency of past behavior regarding physical activity before the pandemic.

### The Present Study

Based on the above literature on the influence of perceived threat and fear on the evaluation of recommendation messages, in the present study we proposed and tested a theoretical model to understand receivers' reactions to gain, non-loss, non-gain and loss messages focused on home-based physical activity. We first measured attitude and intention We examined whether perceived *threat* or *fear* would have an impact on message evaluation, and thus would influence attitude and intention toward home-based physical activity at Time 2 differently, in the case of a recommendation framed as *gain, non-loss, non-gain or loss*.

Given that literature on threat and fear triggered by the four different message frames is scarce, we did not make specific hypotheses about the various relationships among the study variables, but only a series of research questions.

*Research Question 1, RQ1*: To what extent does message-induced *threat* influence message evaluation, attitude and intention regarding home-based activity at Time 2 in the four different message conditions?

*Research Question 2, RQ2*: To what extent does message-induced *fear* influence message evaluation, attitude and intention regarding home-based activity at Time 2 in the four different message conditions?

*Research Question 3, RQ3*: How far *attitude and intention at Time 1, frequency of past behavior and self-efficacy* influence message evaluation, attitude and intention at Time 2 regarding home-based activity in the four different message conditions?

## Method

### Procedure and Participants

In April 2020, a sample of Italian citizens was invited to participate in a university study on public communication. Participants were recruited by students of the Department of Psychology of the Catholic University of the Sacred Heart (Italy), and received an email with a link to an online survey developed through the Qualtrics platform. Through the online survey, participants:

- completed the first part of a questionnaire measuring the psychological antecedents of home-based physical activity (Time 1);
- were then automatically and randomly assigned to four different conditions (gain, non-loss, non-gain, and loss messages) and were invited to read an infographic reporting a series of messages on the physical and psychological consequences of exercising at home;
- after reading the messages, were required to fill in the second part of the questionnaire (Time 2).

The initial sample was made of *N* = 280 participants. Participants who did not fully or accurately complete the questionnaire were then excluded (*N* = 8). So, the final sample consisted of 272 participants (126 males, 142 females, 4 other; mean age = 42.97, *SD* = 14.98, age range = 18–70), distributed in the four message conditions as follows: gain message condition *N* = 70; non-loss message condition *N* = 67; non-gain message condition *N* = 67; loss message condition *N* = 68.

### Pre-test Measures

At the beginning of the questionnaire, participants provided their informed consent and read the following statement: “We are interested in understanding what drives people to do physical exercises at home in the absence of alternatives (i.e., in the impossibility of accessing parks, gyms and open spaces). By physical activity at home we mean, for example: bodyweight workout, such as stretching, aerobics, push-ups, and abs; walking for at least 30 min (6,000 steps per day); training with weights and machines, such as stationary bikes and treadmills.” After that, participants responded to a series of questions aimed at measuring their frequency of past behavior, attitude and intention toward home-based physical activity, and self-efficacy.

*Frequency of past behavior* related to physical activity was measured with 2 items regarding how often participants engaged in physical activity away from home and at home before the lockdown restrictions: “Before this period of restrictions, on average how many times a week did you engage in moderate or intense physical activity outdoor - e.g., fast walking, climbing stairs, cycling, swimming, going to the gym, going for a run etc.?”; “Before this period of restrictions, on average how many times a week did you exercise at home?.” Answers were given on a seven-point Likert scale, from never (1) to every day (7). Higher scores indicated a higher frequency of physical activity before the lockdown restrictions.

*Intention at Time 1* toward doing home-based physical activity was assessed with 3 items on a seven-point Likert scale [completely disagree (1) – completely agree (7)] (e.g., “I intend to do physical exercises at home regularly in the next month”; Clark and Bassett, 2014). Higher scores indicated a greater intention to exercise at home at Time 1.

*Attitude at Time 1* toward home-based physical activity was measured using 5 items on a semantic differential scale ranging from “1” to “7” (e.g., “I believe that doing physical exercises at home regularly is… useless – useful”; Caso et al., 2020). Higher values indicated a more positive attitude toward exercising at home at Time 1.

*Self-efficacy* related to regular physical activity was measured using 6 items on a seven-point Likert scale [completely disagree (1) – completely agree(7)] (e.g., “If I wanted, I would be able to do the physical activity regularly when I am feeling tired”; Bandura, 1977). Higher values indicated a more positive self-efficacy toward exercising at home.

### Message Intervention

After completing the first questionnaire, all participants were invited to read one infographic including 6 messages (~14 words each) describing the physical, mental and social consequences of doing physical activity at home, and formulated in prefactual terms (i.e., “If only…”; see Carfora et al., 2019; Bertolotti et al., 2020). Participants read different messages according to the experimental condition to which they had been randomly assigned. Participants in the *gain message condition* read messages on the positive outcomes associated with doing home-based physical activity (e.g., “If you do physical activity at home, you will improve your fitness.”). Participants in the *non-loss message condition* read messages informing about how doing home-based physical activity relates to preventing negative outcomes (e.g., “If you do physical activity at home, you will avoid worsening your fitness.”). Participants in the *non-gain message condition* read messages emphasizing how doing home-based physical activity is related to missing out positive consequences (e.g., “If you do not do physical activity at home, you will lose the chance to improve your fitness.”). Finally, participants in the *loss message condition* read messages on the negative outcomes of not doing home-based physical activity (e.g., “If you do not do physical activity at home, you will worsen your fitness.”). The full list of messages is reported in Appendix 1 in Supplementary Material.

### Post-test Measures

After reading the messages, participants completed the second part of the questionnaire, which measured the dimensions described below.

*Message-induced fear* was measured with five items pertaining to the degree to which reading messages had made participants feel fearful (e.g., “To what extent reading these messages made you feel scared?”; adapted from Brown and Smith, 2007). Answers were given on a 7-point Likert scale, from (1) “not at all” to (7) “completely.” Higher values indicated higher fear after reading the messages.

*Message-induced threat* was measured with four items related to how much reading messages had made participants feel their freedom threatened (e.g., “The messages have tried to pressure me”; adapted from Shen, 2015). Answers were given on a 7-point Likert scale, from (1) “strongly disagree” to (7) “strongly agree.” Higher values indicated higher perceived threat.

*Message evaluation* was measured with three items asking participants to state how involved they had been in the messages (e.g., “Messages were very interesting”; adapted from Godinho et al., 2016). Answers were given on a 7-point Likert scale, from (1) “strongly disagree” to (7) strongly agree.” Higher values indicated a more positive evaluation of the messages.

Finally, we again measured receivers' attitude and intention toward home-based physical activity at Time 2 after message exposure, using the same scale and items used at Time 1.

At the end of the second part of the questionnaire, participants reported their age and gender.

### Data Analysis

As a first step of our analysis, we assessed the variance inflation factor (VIF) to compute multicollinearity. The VIF results, which are below threshold value of 5.0, indicate that collinearity issues among the study variables is absent (Hair et al., 2016).

Then, we used confirmatory factor analysis to verify the measurement model. To verify the internal consistency among the measurement items for each variable, we used composite reliability. We also tested convergent and discriminant validities among our variables.

The adequacy of fit of the measurement and structural models were estimated using a chi-square test and recommended incremental goodness-of-fit indices: the root mean square error of approximation (RMSEA), the comparative fit index (CFI), and the Tucker-Lewis Index (TLI). A nonsignificant chi-square test indicates that the model fits the data well (Iacobucci, 2010). RMSEA value of 0.05 or less indicates a good fit and values up to 0.10 represent errors that approximate those expected in the population (Iacobucci, 2010). Finally, CFI and TLI cut-off values of at least 0.90 are generally considered to represent an acceptable fit (Iacobucci, 2010).

After confirming the adequacy of fit of our structural model, we used it as a base model to test the invariance of the relationship between study variables across groups. We first applied a *multi-group Structural Equation Modeling (SEM)* to observe the relationships among study variables in each group. We then constrained the main significant paths of each group to be equal in the other groups, while we left the other path coefficients free to vary across groups. By disconfirming the equality (or invariance) of the main significant paths, we would be able to establish that the diverse messages read by participants moderated the relationship among the psychological antecedents of home-based physical activity, the reactions to the messages, and attitude and intention regarding home-based physical activity at Time 2. We evaluated the null hypothesis of the equalities of such paths across message groups through a Wald test.

## Results

### Preliminary Analyses

Table 1 shows means, standard deviations, composite reliability and average variance extracted (AVE) of each study variable, plus standard loadings of each item employed to measure the variable. Table 2 reports the estimates relevant to convergent and discriminant validity.

**Table 1:** Results of the confirmatory factor analysis.

**Construct**	**Mean**	**Standard deviation**	**Items**	**Standard loadings**	**Composite reliability**	**AVE**
Frequency of Past Behavior (PB)	3.51	1.43	PB1	0.68	0.76	0.61
			PB2	0.35		
Intention at Time 1 (INT_T1)	5.15	1.75	INT_T1_1	0.94	0.98	0.94
			INT_ T1_2	0.98		
			INT_ T1_3	0.95		
Attitude at Time 1 (ATT_T1)	5.58	1.63	ATT_T1_1	0.71	0.96	0.82
			ATT_T2_2	0.92		
			ATT_T2_3	0.91		
			ATT_T2_4	0.90		
			ATT_T2_5	0.90		
Self-Efficacy (SE)	3.97	1.57	EFF1	0.70	0.93	0.72
			EFF2	0.83		
			EFF3	0.82		
			EFF4	0.82		
			EFF5	0.86		
Message-Induced Fear (MIF)	1.21	0.43	MIF1	0.71	0.91	0.68
			MIF2	0.75		
			MIF3	0.88		
			MIF4	0.87		
			MIF5	0.70		
Message-Induced Threat (MIT)	2.50	1.28	MIT1	0.71	0.92	0.75
			MIT2	0.86		
			MIT3	0.90		
			MIT4	0.82		
Message Evaluation (ME)	4.92	1.17	ME1	0.92	0.97	0.78
			ME2	0.78		
			ME3	0.75		
Attitude at Time 2 (ATT_T2)	5.79	1.52	ATT_T2_1	0.68	0.95	0.79
			ATT_T2_2	0.94		
			ATT_T2_3	0.91		
			ATT_T2_4	0.93		
			ATT_T2_5	0.85		
Intention at Time 2 (INT_T2)	5.17	1.70	INT_T2_1	0.96	0.97	0.96
			INT_T2_2	0.98		
			INT_T2_3	0.95		

**Table 2:** Convergent and discriminant validity.

	**1**.	**2**.	**3**.	**4**.	**5**.	**6**.	**7**.	**8**.	**9**.
1. Frequency of past behavior	**0.67**	0.30^*^	0.06	0.31^*^	0.17^*^	−0.05	−0.02	0.14^*^	0.29^*^
2. Intention at Time 1		**0.94**	0.38^*^	0.50^*^	−0.00	−0.06	0.26^*^	0.39^*^	0.84^*^
3. Attitude at Time 1			**0.82**	0.29^*^	−0.14^*^	−0.01	0.18^*^	0.76^*^	0.35^*^
4. Self-efficacy				**0.72**	−0.10	−0.04	0.25^*^	0.37^*^	0.52^*^
5. Message-induced fear					**0.68**	0.21^*^	0.07	−0.08	0.02
6. Message-induced threat						**0.75**	−0.31^*^	−0.11	−0.10^*^
7. Message evaluation							**0.78**	0.34^*^	0.37^*^
8. Attitude at Time 2								**0.79**	0.48^*^
9. Intention at Time 2									**0.96**

*The values in the diagonal row (bold) are the average variance extracted by each latent construct. The numbers above diagonal are the correlation coefficients between the constructs*.

* *p < 0.001*.

The VIF results for each dependent variable were below threshold value of 5.0 (message-induced threat = 1.00; message-induced fear = 1.03; message evaluation = 1.30; attitude at Time 2 = 2.80; intention at Time 2 = 4.13). This result indicated that collinearity issues among the study variables were absent from this study.

Confirmatory factor analysis showed that the measurement model fit the data satisfactorily ($\chi_{\left(2\right)}^{2}$ = 3.58, *p* = 0.17; RMSEA = 0.05, CFI = 0.99, TLI = 0.97, SRMR = 0.02). Results revealed that all the composite reliability values were greater than the minimum threshold of 0.60 (Bagozzi and Yi, 1988), ranging from 0.76–0.98. Thus, the reliability of the measurement model was confirmed. The standardized item loadings of all observed variables on their corresponding latent constructs varied from 0.68–0.98 (Table 1), except for one of the two items measuring frequency of past behavior. Thus, standardized item loadings were mainly significant. The AVE from latent constructs ranged from 0.61 to 0.96. Therefore, all AVE values were above the recommended threshold of.50 (Anderson and Gerbing, 1988). These findings showed that all measurement items presented a high convergent validity. Discriminant validity was also confirmed, because all AVEs were higher than squared correlations between latent constructs (Fornell and Larcker, 1981). Finally, we confirmed the adequacy of fit of our structural model ($\chi_{\left(524\right)}^{2}$ = 1018.51, *p* = 0.001; RMSEA = 0.03, CFI = 0.94, TLI = 0.94, SRMR = 0.05).

### Main Analyses

#### Multi-Group SEM Model

In the main analyses, we used the tested model to disconfirm the null hypothesis of the invariance of the relationships among the study variables across groups. We did so by computing a multi-group SEM model with the message groups. The goodness-of-fit statistics for the model were acceptable. The chi-square test was not significant (χ^2^ = 13.78, *df* = 8, *p* = 0.09) and also the other indices pointed to an acceptable fit (*RMSEA* = 0.10; *CFI* = 0.99; *TLI* = 0.90; χ^2^ gain message group = 3.34; χ^2^ loss message group = 7.20; χ^2^ non-gain message group = 0.01; χ^2^ non-loss message group = 3.22), indicating that dataset had overall a good model fit.

We then analyzed the parameter estimates of the model in the four message conditions (gain, non-loss, non-gain, loss). All parameter estimates are reported in Appendix 3 in Supplementary Material. Below, we will consider the predictors of all dependent variables related to our three main research questions, namely, how message-induced threat predicted message evaluation, attitude and intention (RQ1), how message-induced fear predicted message evaluation, attitude and intention at Time 2 (RQ2), and how the psychological antecedents of home-based activity influenced message evaluation, attitude and intention at Time 2 (RQ3).

**Figure 1:**
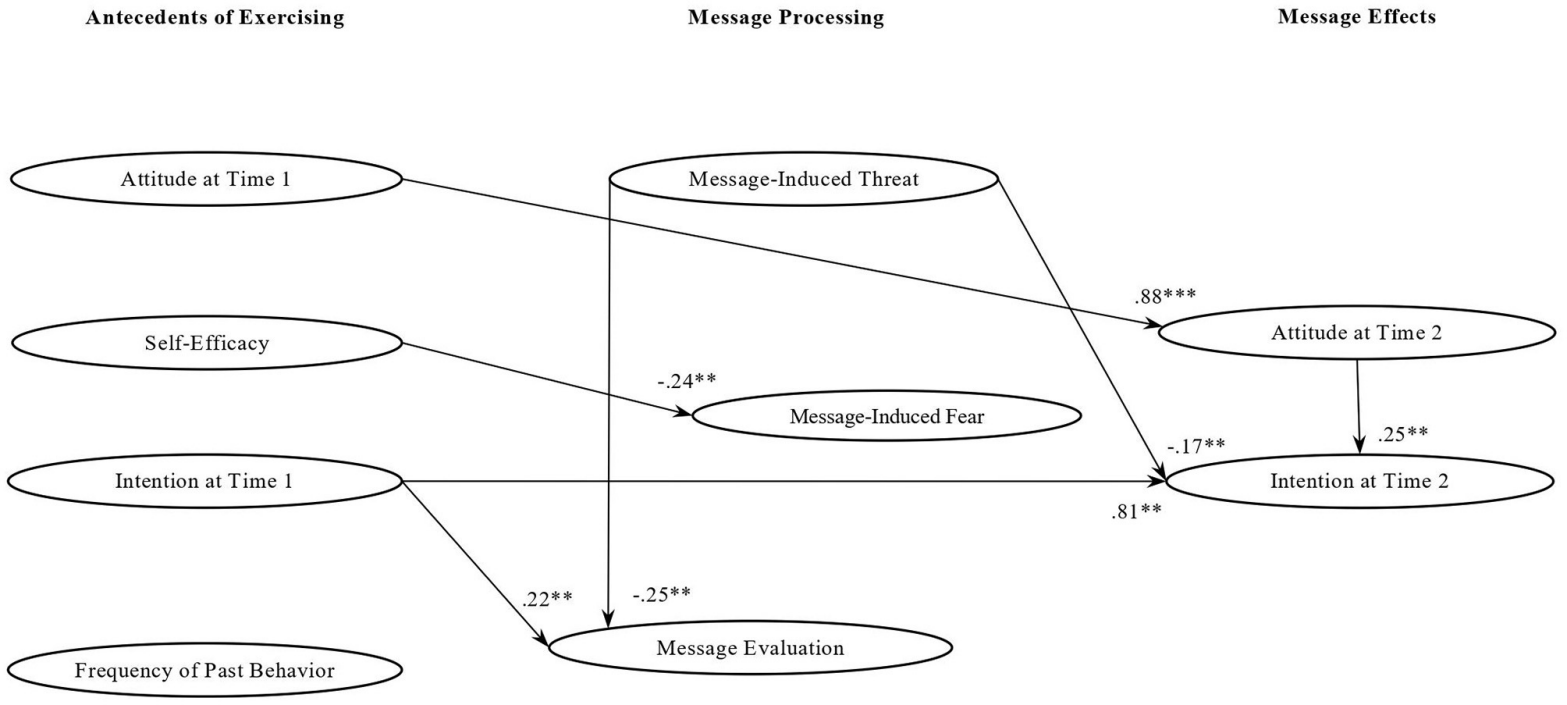
Standardized factor loadings of the relationships among study variables in the gain message group. **p < 0.05; ***p < 0.001.

As showed in Figure 1, when participants were exposed to *gain messages* the perception that the messages were not threatening increased the positive evaluation of the messages (β = −0.25; *p* = 0.04), as well as the intention to do home-based physical activity at Time 2 (β = −0.17; *p* = 0.05). Message-induced fear did not predict message evaluation, attitude at Time 2, or intention at Time 2, but a high level of self-efficacy reduced the perception of the gain messages as being fearful (β = −0.24; *p* = 0.05). Positive attitude at Time 1 had a direct effect on positive attitude at Time 2 (β = 0.88; *p* = 0.001), and in turn attitude at Time 2 determined a higher intention to exercise at home at Time 2 (β = 0.25; *p* = 0.05). Actually, the effect of attitude at Time 1 on intention at Time 2 was fully mediated by attitude at Time 2 (*Ind*. = *0.2*2; *p* = 0.05). When participants had higher intention to do physical activity before message exposure they also gave a more positive evaluation of the gain messages (β = −0.22; *p* = 0.05) and had higher intention at Time 2 (β = 0.81 *p* = 0.05). To sum up, these results showed that gain messages had an impact on intention at Time 2 mainly because this message frame was not perceived as threatening. Moreover, there was an increase in intention at Time 2 especially when participants had a positive attitude toward home-based physical activity both at Time 1 and Time 2.

**Figure 2:**
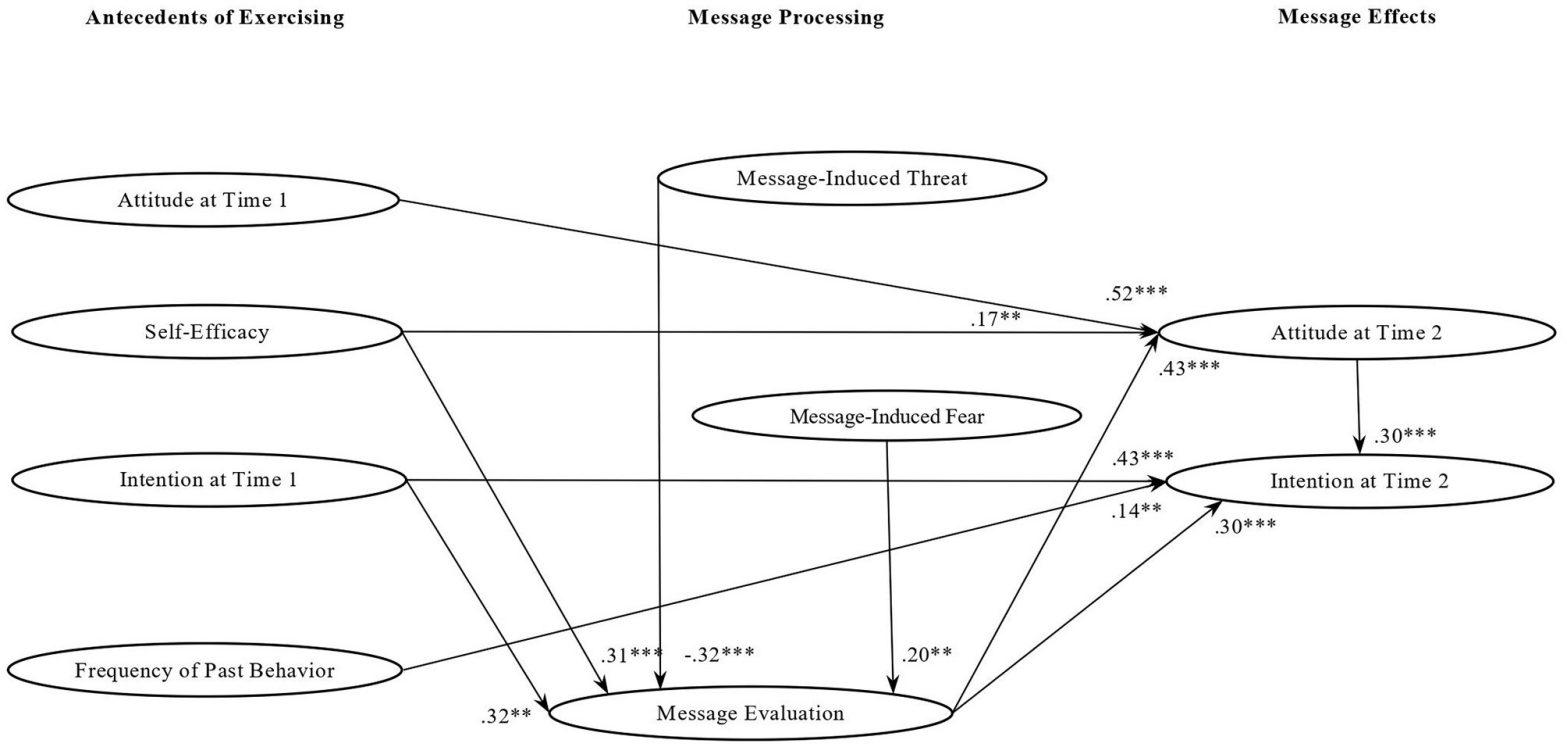
Standardized factor loadings of the relationships among study variables in the non-loss message group. **p < 0.05; ***p < 0.001.

In the case of participants exposed to *non*-*loss messages* (Figure 2), the perception of the messages as not threatening predicted a positive message evaluation (β = −0.32; *p* = 0.001), which in turn influenced attitude at Time 2 (β = 0.43; *p* = 0.001) and then intention at Time 2 (β = 0.30; *p* = 0.001). Positive message evaluation also had a direct effect on intention at Time 2 (β = 0.30; *p* = 0.001). Consistently, mediation analyses confirmed that the negative impact of threat on intention at Time 2 was fully mediated by the participants' positive evaluation of the messages (*Ind*. = −0.11; *p* = 0.01) and by the effect of this positive evaluation on attitude at Time 2 (*Ind*. = −0.05; *p* = 0.03). Moreover, in this group message-induced fear increased a positive message evaluation (β = 0.20; *p* = 0.05), which in turn marginally increased attitude at Time 2 and then intention at Time 2 (*Ind*. = −0.06; *p* = 0.10). As to the other antecedents of physical activity, a higher level of self-efficacy predicted both a more positive message evaluation (β = 0.31; *p* = 0.001) and a higher attitude at Time 2 (β = 0.17; *p* = 0.05). Moreover, mediation results showed that receivers' with higher self-efficacy had higher intention to exercise at home at Time 2 thanks to the effect of a more positive message evaluation (*Ind*. = *0.1*3; *p* = 0.01) on their attitude at Time 2 (*Ind*. = *0.0*6; *p* = 0.02). Attitude at Time 1 had a direct effect on participants' attitude at Time 2 (β = 0.52; *p* = 0.001) and an indirect effect on intention at Time 2 that was fully mediated by attitude at Time 2 (*Ind*. = *0.1*6; *p* = 0.05). In addition, intention at Time 1 (β = 0.43; *p* = 0.001) and frequency of past behavior (β = 0.14; *p* = 0.03) determined receivers' intention to do home-based physical activity at Time 2. To sum up, these results showed that non-loss messages were effective in increasing intention at Time 2 when the messages were perceived as not threatening, but triggered some fear, especially when participants had a high self-efficacy.

**Figure 3:**
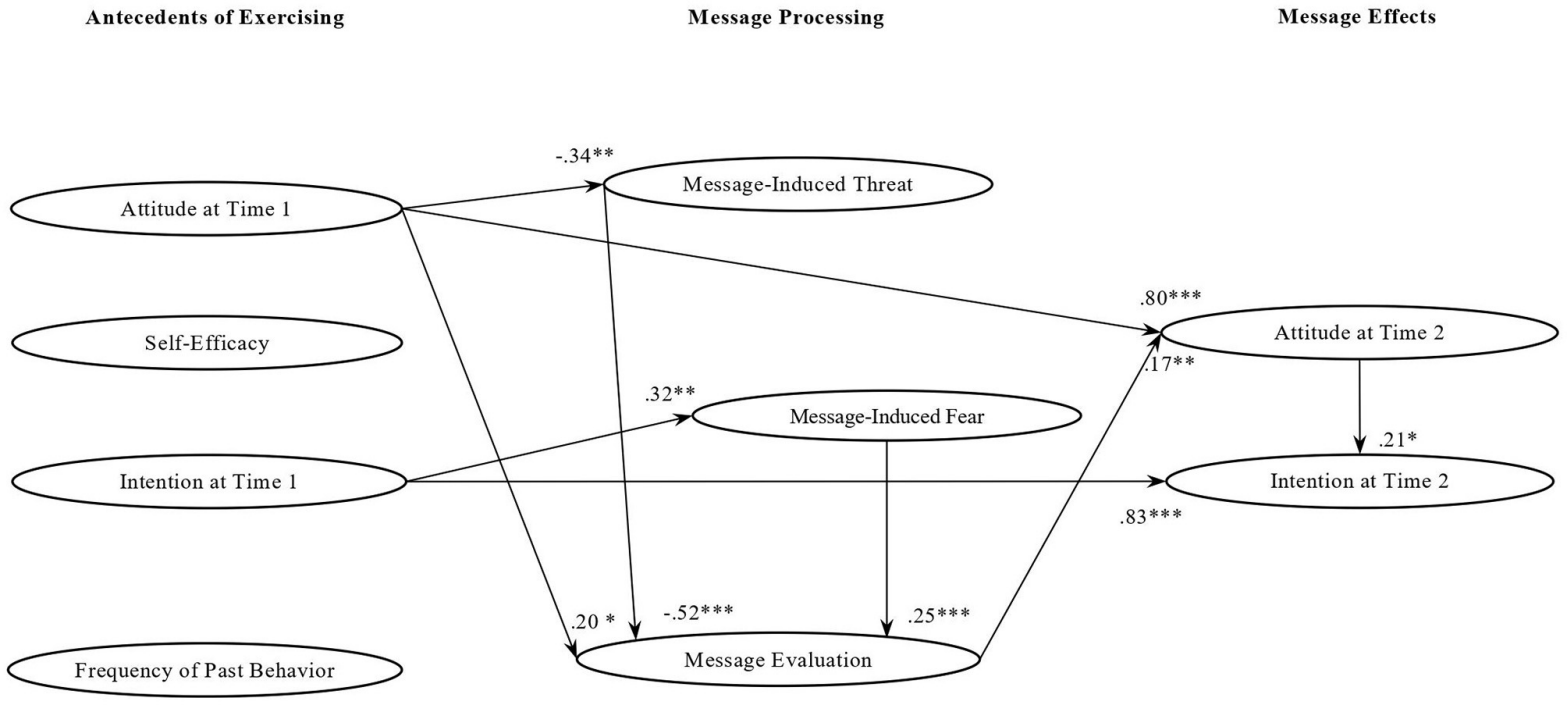
Standardized factor loadings of the relationships among study variables in the non-gain message group. *p < 0.10; **p < 0.05; ***p < 0.001.

In the case of participants exposed to *non-gain messages* (Figure 3), a higher perception that the messages were not threatening determined a more positive message evaluation (β = −0.54; *p* = 0.001), and mediation analyses showed that there was also an indirect effect of message-induced threat on attitude at Time 2 through message evaluation (*Ind*. = −0.09; *p* = 0.03). As in the case of non-loss messages, also in the case of non-gain messages a higher perception that the messages were fearful increased the positive evaluation of the messages (β = 0.25; *p* = 0.001) and the impact of message-induced fear on attitude at Time 2 was mediated by message evaluation (*Ind*. = *0.0*9; *p* = 0.05). A more positive evaluation of the messages increased attitude at Time 2 (β = 0.16; *p* = 0.02), which in turn marginally influenced intention at Time 2 (β = 0.21; *p* = 0.10). As to the influence of baseline variables, a higher attitude at Time 1 influenced their attitude at Time 2 (β = 0.80; *p* = 0.001), strongly decreased perceived message-induced threat (β = −0.34; *p* = 0.001), and increased a positive message evaluation (β = 0.20; *p* = 0.03). This chain was also marginally confirmed by a mediation analysis (*Ind*. = 0.03; *p* = 0.10). In turn, a higher level of intention at Time 1 influenced intention at Time 2 (β = 0.83; *p* = 0.001), but it also increased message-induced fear (β = 0.32; *p* = 0.001). However, the indirect impact of intention at Time 1 on attitude at Time 2 through message-induced fear was only marginally confirmed (β = 0.04; *p* = 0.08). To sum up, these findings indicated that in the case of non-gain messages the impact of message processing on attitude and intention at Time 2 was rather limited. Intention at Time 2 was only marginally predicted by attitude at Time 2, which in turn was only marginally influenced by message evaluation.

**Figure 4:**
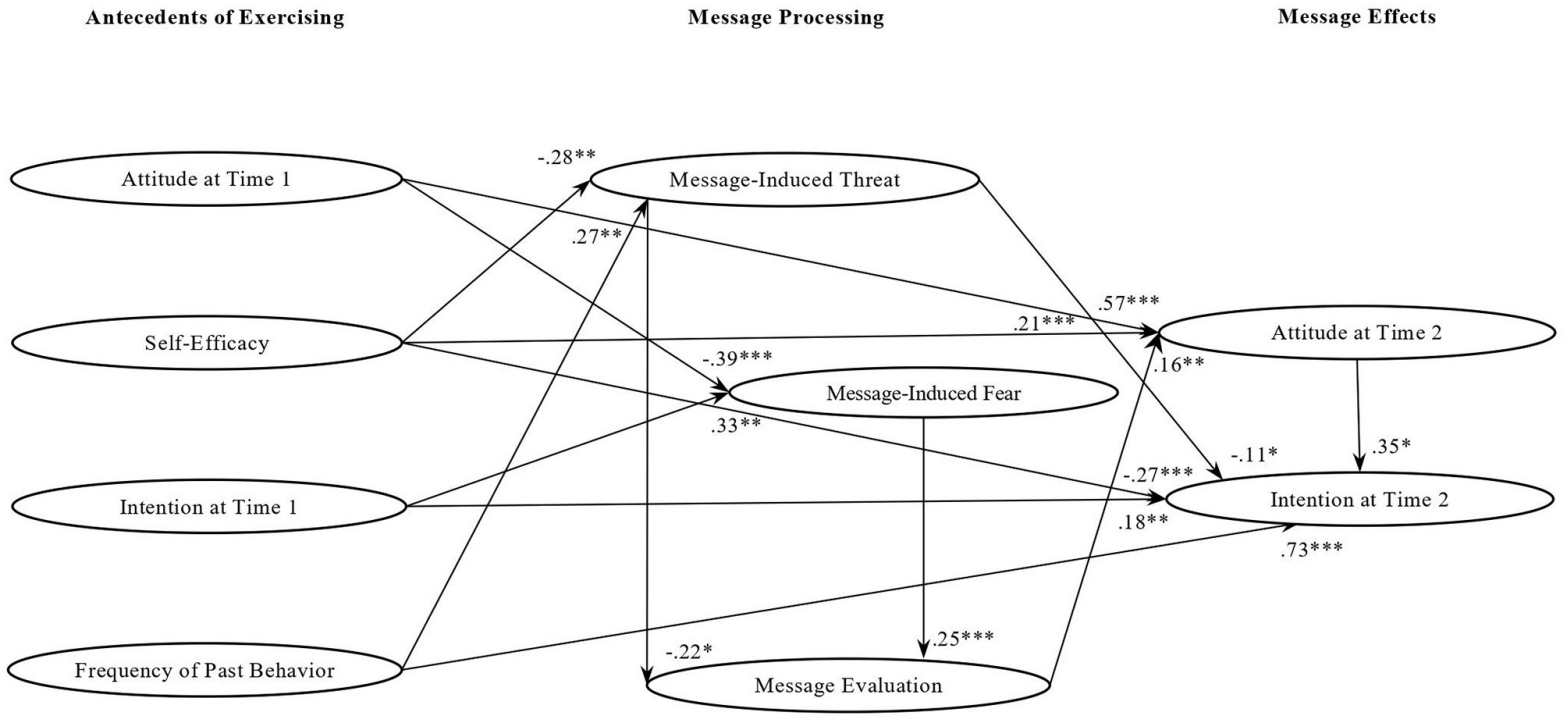
Standardized factor loadings of the relationships among study variables in the loss message group. *p < 0.10; **p < 0.05; ***p < 0.001.

Finally, in the case of participants exposed to *loss messages* (Figure 4), message-induced threat had a marginal effect both on message evaluation (β = −0.22; *p* = 0.07) and intention at Time 2 (β = −0.11; *p* = 0.08). As to message-induced fear, it stimulated a positive message evaluation (β = 0.25; *p* = 0.04). A more positive evaluation of the messages increased attitude at Time 2 (β = 0.16; *p* = 0.05), which in turn influenced intention to do physical activity at home at Time 2 (β = 0.35; *p* = 0.001). In this message group, positive attitude at Time 1 (β = 0.57; *p* = 0.001) increased attitude at Time 2 and had an indirect effect on intention at Time 2 through attitude at Time 2 (*Ind*. = *0.2*0; *p* = 0.001). However, loss messages were counterproductive for people with a high level of positive attitude at Time 1, who did not perceive the messages as fearful (β = −0.39; *p* = 0.001) and showed a lower intention at Time 2 after reading these messages (β = −0.27; *p* = 0.001). A higher intention at Time 1 predicted both a higher intention at Time 2 (β = 0.73; *p* = 0.001) and a higher message-induced fear (β = 0.33; *p* = 0.001). Instead, participants with high self-efficacy perceived the loss messages as less threatening (β = −0.28; *p* = 0.05) and had a more positive attitude at Time 2(β = 0.21; *p* = 0.04) and intention (β = 0.18; *p* = 0.02) toward home-based physical activity. Self-efficacy had also a positive indirect effect on intention at Time 2 via attitude at Time 2 (*Ind*. = *0.0*7; *p* = 0.05). Regarding the role of the frequency of past behavior, people with high frequency of past behavior perceived messages as more threatening (β = 0.27; *p* = 0.03). However, there was not a significant mediation effect from frequency of past behavior to intention at Time 2. To sum up, the perception and the consequences of loss messages were differently affected by the baseline antecedents of physical activity. If a high level of self-efficacy increased their persuasiveness, a high level of attitude at Time 1 and frequency of past behavior decreased it.

#### Determination of Invariant Paths in the Multigroup SEM Model

To disconfirm the null hypothesis of the invariance of the main significant paths among study variables across groups, we then used the Wald test. Table 3 reports all the Wald tests for each comparison.

**Table 3:** Results of the comparisons of the main significant paths among message groups.

	**Gain vs. non-loss messages**	**Gain vs. non-gain messages**	**Gain vs. loss messages**	**Non-loss vs. non-gain messages**	**Non-loss vs. loss messages**	**Non-gain vs. loss messages**
a. Message-Induced Threat → Intention at Time 2	$\chi_{\left(1\right)}^{2}$ = 5.08 *p* = 0.02	*χ^2^*_(1)_ = 6.03 *p* = 0.01	$\chi_{\left(1\right)}^{2}$ = 10.07 *p* = 0.001	/	/	/
b. Message-Induced Threat → Message Evaluation → Attitude at Time 2 → Intention at Time 2	$\chi_{\left(1\right)}^{2}$ = 9.11 *p* = 0.002	/	/	/	$\chi_{\left(1\right)}^{2}$ = 4.54 *p* = 0.03	/
c. Self-Efficacy → Message Evaluation → Intention at Time 2	$\chi_{\left(1\right)}^{2}$ = 5.09 *p* = 0.02	/	/	$\chi_{\left(1\right)}^{2}$ = 6.15 *p* = 0.01	$\chi_{\left(1\right)}^{2}$ = 6.89 *p* = 0.01	/
d. Message-Induced Fear → Message Evaluation → Attitude at Time 2 → Intention at Time 2	$\chi_{\left(1\right)}^{2}$ = 2.79 *p* = 0.09	*/*	/	$\chi_{\left(1\right)}^{2}$ = 1.77 *p* = 0.18	$\chi_{\left(1\right)}^{2}$ = 2.79 *p* = 0.09	*/*
e. Self-Efficacy → Attitude at Time 2 → Intention at Time 2	$\chi_{\left(1\right)}^{2}$ = 1.99 *p* = 0.16	/	$\chi_{\left(1\right)}^{2}$ = 2.79 *p* = 0.05	$\chi_{\left(1\right)}^{2}$ = 2.51 *p* = 0.11	$\chi_{\left(1\right)}^{2}$ = 0.21 *p* = 0.64	$\chi_{\left(1\right)}^{2}$ = 3.26 *p* = 0.05
f. Self-Efficacy → Message Evaluation → Attitude at Time 2 → Intention at Time 2	$\chi_{\left(1\right)}^{2}$ = 0.77 *p* = 0.38	/	/	$\chi_{\left(1\right)}^{2}$ = 1.65 *p* = 0.19	$\chi_{\left(1\right)}^{2}$ = 3.50 *p* = 0.05	/
g. Message-Induced Fear → Message Evaluation	$\chi_{\left(1\right)}^{2}$ = 1.04 *p* = 0.31	$\chi_{\left(1\right)}^{2}$ = 2.21 *p* = 0.14	/	$\chi_{\left(1\right)}^{2}$ = 0.38 *p* = 0.53	$\chi_{\left(1\right)}^{2}$ = 0.04 *p* = 0.84	$\chi_{\left(1\right)}^{2}$ = 0.75 *p* = 0.39
h. Message-Induced Threat → Message Evaluation → Attitude at Time 2	/	$\chi_{\left(1\right)}^{2}$ = 4.25 *p* = 0.03	*/*	$\chi_{\left(1\right)}^{2}$ = 0.46 *p* = 0.50	/	$\chi_{\left(1\right)}^{2}$ = 1.88 *p* = 0.17
i. Attitude at Time 1 → Message-Induced Threat → Message Evaluation → Attitude at Time 2	/	$\chi_{\left(1\right)}^{2}$ = 5.30 *p* = 0.02	*/*	$\chi_{\left(1\right)}^{2}$ = 5.06 *p* = 0.02	/	$\chi_{\left(1\right)}^{2}$ = 3.64 *p* = 0.05
j. Frequency of Past Behavior → Message-Induced Threat	/	/	$\chi_{\left(1\right)}^{2}$ = 0.93 *p* = 0.33	/	$\chi_{\left(1\right)}^{2}$ = 4.65 *p* = 0.03	$\chi_{\left(1\right)}^{2}$ = 3.39 *p* = 0.05

Compared to the other message conditions, only in the gain message condition perceiving the messages as not threatening directly increased intention at Time 2 to do home-based physical activity (Table 3, a). Instead, in the non-loss message condition receivers perceiving the messages as not threatening evaluated them more positively, as increased their attitude and intention at Time 2 more as compared to receivers in the other message conditions (Table 3, b). Moreover, only in the case of the non-loss messages, when receivers perceived themselves as being able to exercise regularly (self-efficacy), they evaluated the messages positively and thus increased intention at Time 2 (Table 3, c). Finally, the self-efficacy-attitude at Time 2-intention at Time 2 chain (Table 3, e), and the self-efficacy-message evaluation-attitude at Time 2-intention at Time 2 chain (Table 3, f) were invariant across all message groups.

The pattern from message-induced fear to message evaluation was invariant across groups (Table 3, g). In addition, Wald tests showed that when receivers perceived the non-gain messages as not threatening, they had more positive evaluation and then more attitude at Time 2, compared to receivers in the gain message (Table 3, h). Wald tests also showed that these higher effect of non-gain message as compared to gain message on attitude at Time 2, via a lower message-induced threat, was even more accentuated when receivers had a high attitude at Time 1 (Table 3, i). Finally, both in the non-loss and non-gain message groups, an effect of message-induced fear on intention at Time 2 through message evaluation and attitude at Time 2 emerged. Wald tests showed that this mediation path was stronger in the non-gain message group than in the non-loss message group (Table 3, d). This result confirmed a high impact of the perception of fear on receivers' message elaboration when exposed to the gain messages.

## Discussion

First of all, our findings confirmed that message-induced threat and fear have an important role in determining the effects of recommendation messages in the context of the promotion of home-based physical activity. Results showed that the persuasiveness of the *gain-framed messages* is based on their being perceived as *not* threatening, so that this perception increases intention to do home-based physical activity at Time 2. This suggests that the major strength of gain-framed messages is their positive valence, which does not stimulate a sense of threat in receivers. In the elaboration of gain-framed messages, message-induced fear plays no significant role, and this absence may be counterproductive, given that messages evoking fear lead people to rely on systematic processing, which in turn stimulates many issue-relevant thoughts, including a positive evaluation of the message (e.g., Meijnders et al., 2001; Slater et al., 2002; Meyers-Levy and Maheswaran, 2004). In consideration of the above, gain-framed messages seem to have an immediate effect because the absence of a threat induces a greater intention to exercise. This effect, however, is not based on systematic processing and belief change (favored by message-induced fear) and is therefore likely to be short-term.

*Non-loss-framed messages* are also perceived as not threatening. However, in this case such perception stimulates a positive evaluation of the message which, in turn, influences attitude and intention at Time 2. Besides, unlike gain-framed messages, non-loss-framed messages stimulate a link from the perception of fear to attitude and intention at Time 2 through a positive message evaluation. This effect can be attributed to loss aversion, the most considered cause of the persuasive effect of the loss frame (O'Keefe, 2012). Loss aversion is a phenomenon related to the fact that people generally prefer to avoid losses rather than obtain gains. In the case of the non-loss frame, the effect of message-induced fear is marginal, and this suggests that this frame does not induce excessive fear, which may lead people to enact defensive strategies to reduce the potential emotional distress associated with the messages (Witte, 1992; Ruiter et al., 2001). Lack of threat and some presence of fear are likely to have contributed to the clear link among message evaluation, attitude and intention at Time 2 observed in receivers exposed to non-loss-framed messages. This strength of the non-loss frame could lie in the fact that it combines the positive aspects of both gain and loss frames. Like the gain frame, the non-loss frame produces a low perceived threat to freedom (Cho and Sands, 2011), that may reduce the psychological reactance. At the same time, proposing the avoidance of negative outcomes, the non-loss frame directs the attention to the possible negative consequences of one's behavior and triggers some fear. Relying on a negative bias, the acquisition of negative information requires greater information processing than does positive information (Rozin and Royzman, 2001). Thus, people tend to think and reason more about non-loss- than gain-framed messages. A greater elaboration may then induce a greater attitude and intention change.

As in the case of gain- and non-loss-framed messages, also in the case of *non-gain-framed messages* the absence of message-induced threat is fundamental for the positive evaluation of the message. However, the positive evaluation of non-gain-framed messages also depends on their perception as fearful, which in turn influences attitude at Time 2 via a higher message evaluation. These effects trigged by message processing do not extend to intention at Time 2, however, and the absence of a strong attitude-intention link could compromise the likelihood of an actual behavioral change. This can be because a recommendation based on missing the chance to obtain positive outcomes may be rather difficult to understand. Thus, in this case the elaboration of the recommendation could exceeds the receivers' processing capacity, which in turn would create an information overload that reduces the quality of the decision.

Finally, the perception of *loss-framed messages* as threatening or fearful does not directly influence message evaluation, attitude and intention at Time 2. Actually, the persuasiveness of loss-framed messages is strongly influenced by the level of self-efficacy of the receivers. When they have high self-efficacy, they have greater attitude at Time 2, and then intention at Time 2. In the case of non-loss-framed messages, these receivers have also a more positive evaluation of the messages. This suggests that both loss- and non-loss-framed messages may be more suitable for those who perceive a high capacity of exercising regularly. These findings confirm the role of self-efficacy in influencing message effects, already established by research on framing effects in other types of recommendation messages (e.g., Bertolotti et al., 2020). Specifically, past studies showed that people who feel that they have the necessary skills to perform message recommendations are more motivated to accept a loss frame and more inclined to change their behavior accordingly (Cauberghe et al., 2009; Riet et al., 2010; Tudoran et al., 2012). Conversely, people who feel they are not able to deal with the requests tend to activate defense mechanisms that lead them to reject the threatening loss message. In the present study, we reported a first evidence that self-efficacy is also an important predictor of how people elaborate non-loss-framed messages.

Our research has several limitations. First, our sample was small and restricted to Italian people, thus the data may not be generalized to other countries. Second, our research design lacked a measure for assessing future behavior and did not include a measure of the volume or amount of past physical activity. Third, we cannot exclude the risk of self-selection bias, as participants were invited for a study on public communication. Finally, participants were exposed only once to short messages on physical activity outcomes, thus we were able to assess only small and short-term effects. Messages delivered over a longer time span and with repeated exposure (e.g., Caso and Carfora, 2017; Carfora et al., 2018) could yield larger and long-term effects on recipients' attitudes and intentions. In sum, future research should carefully retest our results on the mechanisms involved in processing messages on physical activity formulated with different frames, sending messages over a longer period. Once said that, the results of the present study can have some useful implications regarding how to select message framing in their communication to promote home-based physical activity in the case of future outbreaks or in other eventualities that require physical exercise at home, such as in the case of rehabilitation programs.

## Conclusion

To sum up, in the present study we validated a model explaining how messages differing according to the outcome sensitivities level of message framing (i.e., gain, non-loss, non-gain and loss messages; Cesario et al., 2013), influence receivers' evaluation of the messages, as well as attitude and intention toward home-based physical activity at Time 2. Our results respond to the need of theoretical advancement in the area of the underlying mechanisms elicited by message framing and show the plausibility of a model including both threat and fear elicited by message exposure. The present study showed that a low perception of threat to freedom strongly contributed to the persuasive effect of the gain and non-loss messages. Moreover, the non-loss messages induced a marginal fear, which may have led participants to systematically process the recommendation but not to enact defensive strategies to reduce a to high emotional distress (Witte, 1992; Ruiter et al., 2001). Instead, when reading loss and non-gain messages, receivers' reactions were more determined by self-efficacy, ending up with reduced persuasive power.

In conclusion, our study introduced and tested an inclusive reference model to explain the effects of message frames based on the presence/absence of positive/negative outcomes of expected behavior and aimed at changing the attitude and intention of the receivers at Time 2. It will be up to future research to further investigate the possibility of applying this model to messages aimed at modifying attitudes and intentions other than the one investigated here, as well as verifying if and how the differences in the mechanisms studied here also depend on individual differences among receivers.

## Data Availability Statement

The raw data supporting the conclusions of this article will be made available by the authors, without undue reservation.

## Ethics Statement

The studies involving human participants were reviewed and approved by Ethics committee of the Department of Psychology - Catholic University of the Sacred Heart - Milan. The patients/participants provided their written informed consent to participate in this study.

## Author Contributions

All authors listed have made a substantial, direct and intellectual contribution to the work, and approved it for publication.

## Conflict of Interest

The authors declare that the research was conducted in the absence of any commercial or financial relationships that could be construed as a potential conflict of interest.
