# Supplementary material for: The Effect of Persuasive Messages in Promoting Home-Based Physical Activity During COVID-19 Pandemic
Source: Front Psychol. 2021 Apr 1;12:644050. doi: 10.3389/fpsyg.2021.644050 (PMC8047668; doi:10.3389/fpsyg.2021.644050)

Appendix 2

Infographics Proposed in the Non-Gain-Framed (on the left) and the Loss-Framed (on the right) Message Conditions

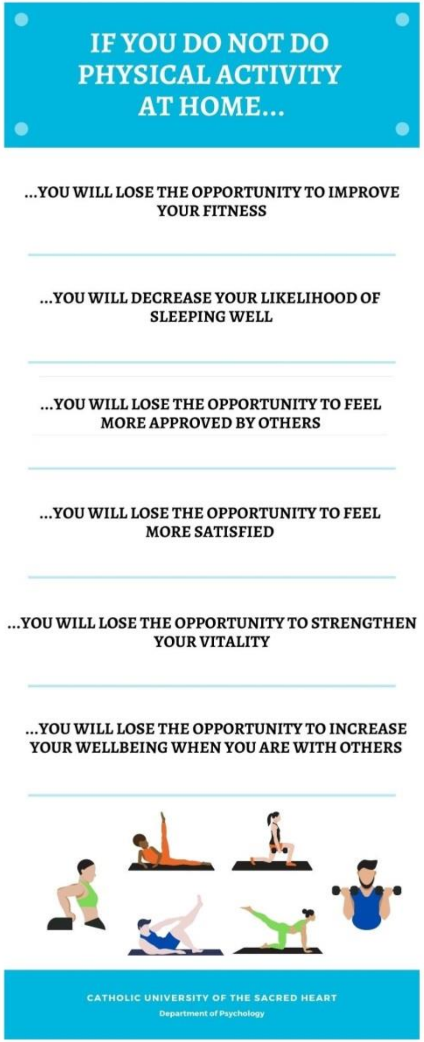

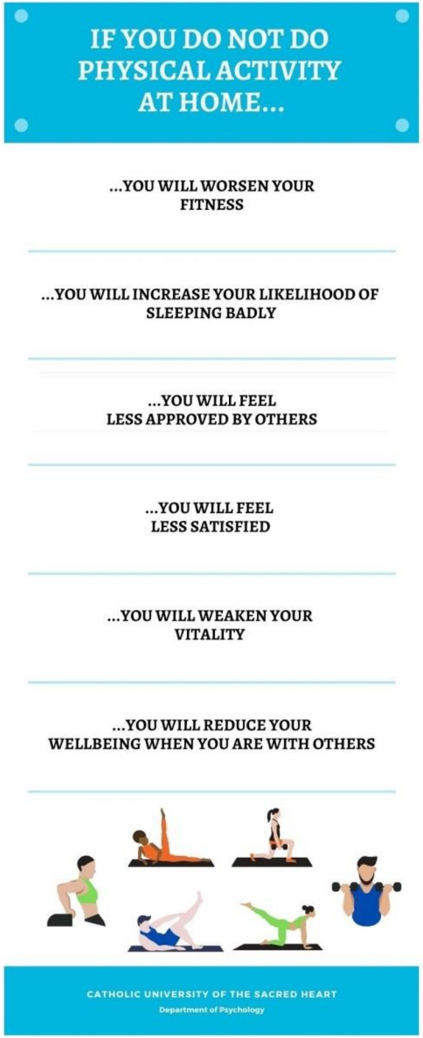

Supplement: Supplementary file 2 [file Image_2.PDF]
