# Supplementary material for: The Effect of Persuasive Messages in Promoting Home-Based Physical Activity During COVID-19 Pandemic
Source: Front Psychol. 2021 Apr 1;12:644050. doi: 10.3389/fpsyg.2021.644050 (PMC8047668; doi:10.3389/fpsyg.2021.644050)
Supplement: Supplementary file 3 [file Table_1.DOCX]

Supplementary Material

**Appendix 3**

*Standardized Factor Loadings Among Message Group Conditions*

|  | Gain Message Group | Non-Loss Message Group | Non-Gain Message Group | Loss Message Group |
| --- | --- | --- | --- | --- |
| Attitude at Time 1→ Message-Induced Threat | -.07 | .15 | -**.34^**^** | .10 |
| Self-Efficacy → Message-Induced Threat | -.01 | .19 | -.09 | **-.28^**^** |
| Intention at Time 1→ Message-Induced Threat | .08 | -.23 | .07 | -.06 |
| Frequency of Past Behavior → Message-Induced Threat | .10 | -.13 | -.06 | **.27^**^** |
|  |  |  |  |  |
| Attitude at Time 1→ Message-Induced Fear | -.06 | .07 | -.18 | **-.39^***^** |
| Self-Efficacy → Message-Induced Fear | -.24 | -.05 | -.11 | -.18 |
| Intention at Time 1→ Message-Induced Fear | -.00 | -.15 | **.32^**^** | **.33^**^** |
| Frequency of Past Behavior → Message-Induced Fear | .17 | -.06 | -.19 | .00 |
|  |  |  |  |  |
| Message-Induced Threat → Message Evaluation | **-.25^**^** | **-.32^***^** | **-.52^***^** | **-.22^*^** |
| Message-Induced Fear → Message Evaluation | .05 | **.20^**^** | **.25^***^** | **.25^***^** |
| Attitude at Time 1 → Message Evaluation | .11 | -.08 | **.20 ^*^** | .12 |
| Self-Efficacy → Message Evaluation | .08 | **.31^***^** | .15 | .15 |
| Intention at Time 1 → Message Evaluation | .22 | **.32^**^** | .01 | -.13 |
| Frequency of Past Behavior → Message Evaluation | .03 | -.03 | -.02 | -.04 |
|  |  |  |  |  |
| Message-Induced Threat → Attitude at Time 2 | .09 | -.09 | -.03 | -.13 |
| Message-Induced Fear → Attitude at Time 2 | .02 | .05 | .05 | .15 |
| Message Evaluation → Attitude at Time 2 | -.01 | **.43^***^** | **.17^**^** | **.16^**^** |
| Attitude at Time 1→ Attitude at Time 2 | **.88^***^** | **.52^***^** | **.80^***^** | **.57^***^** |
| Self-Efficacy → Attitude at Time 2 | .01 | **.17^**^** | -.01 | **.21^***^** |
| Intention at Time 1 → Attitude at Time 2 | -.02 | .09 | -.00 | .20 |
| Frequency of Past Behavior → Attitude at Time 2 | .06 | -.07 | .05 | .02 |
|  |  |  |  |  |
| Message-Induced Threat → Intention at Time 2 | **-.17^**^** | .03 | .07 | **-.11^*^** |
| Message-Induced Fear → Intention at Time 2 | .07 | -.05 | .04 | -.06 |
| Message Evaluation → Intention at Time 2 | .09 | **.30^***^** | .02 | -.06 |
| Attitude at Time 2→ Intention at Time 2 | **.25^**^** | **.30^***^** | **.21^*^** | **.35^*^** |
| Attitude at Time 1→ Intention at Time 2 | -.16 | .03 | -.13 | **-.27^***^** |
| Self-Efficacy → Intention at Time 2 | .06 | .05 | .04 | **.18^**^** |
| Intention at Time 1→ Intention at Time 2 | **.81^**^** | **.43^***^** | **.83^*^** | **.73^***^** |
| Frequency of Past Behavior → Intention at Time 2 | -.02 | **.14^**^** | .04 | -.07 |

*Note. *p* < .10; ***p* < .05; ****p* < .001.
